# Timeliness and Quality of Senior Medic Reviews in Acute Psychiatric Inpatient Care: A Retrospective MultiWard Audit

**DOI:** 10.1192/bjo.2026.11722

**Published:** 2026-06-30

**Authors:** Farhad Farid Hosseini, Naga Gollapenne, Astha Astha, Sabbir Chowdhury, Saad Sarwar

**Affiliations:** Tees, Esk & Wear Valley NHS Trust, Middlesbrough, United Kingdom

## Abstract

**Aims:**

Senior medical reviews are essential for safe inpatient psychiatric care, supporting early risk assessment, treatment planning, and multidisciplinary team (MDT) coordination. National standards, including the Royal College of Psychiatrists’ *Standards for Inpatient* *Mental Health Services – Fourth Edition (2022)* and NHS England’s guidance on *Acute Inpatient Mental Health Care for Adults and Older Adults*, emphasise timely consultant involvement, structured review intervals, and high-quality documentation. This audit aimed to evaluate (1) whether senior medic reviews–conducted by consultants, specialty registrars (SRs), specialty and associate specialist (SAS) doctors, and multi-professional Approved Clinicians (ACs)–were completed within expected timeframes; (2) adherence to required review intervals; and (3) the quality and completeness of clinical documentation.

**Methods:**

A retrospective audit of electronic clinical records was undertaken for psychiatric inpatients admitted to four acute wards at Roseberry Park Hospital during April 2025. Patients admitted for at least 24 hours were included. A structured audit tool assessed senior medic review within 24 hours of admission, completion of 72-hour formulation meetings and discharge reviews, documentation of senior presence in MDT meetings, integration of MDT recommendations, risk assessment, medication monitoring, and statutory documentation for detained patients. Percentages were rounded to the nearest whole number.

**Results:**

Senior medic assessment within 24 hours of admission occurred in 86%. Reviews at expected intervals were completed in 76%. Senior presence in key MDT meetings, integration of MDT recommendations, documentation of risk factors, contingency planning, and statutory requirements for detained patients all achieved full compliance. Documentation of senior medic reviews was clearly recorded in 80%. Clinical entries included diagnosis, risk assessment, treatment decisions, and progress updates in 97%. Medication adherence and side-effect monitoring were documented in 83%. Discharge planning was recorded in 97%. No criteria fell below the red compliance threshold. Areas requiring improvement included delayed initial reviews, inconsistent scheduling of review intervals, and incomplete documentation of progress reviews.

**Conclusion:**

Overall compliance with national standards for senior medic reviews was high, with several domains achieving full adherence. The findings demonstrate strong MDT integration, robust risk documentation, and consistent statutory compliance. However, delays in initial senior reviews, variability in review scheduling, and gaps in documentation highlight opportunities for improvement. Strengthening administrative monitoring, introducing structured review checklists, and reinforcing documentation standards may enhance consistency, improve alignment with national expectations, and support safer inpatient care.

